# Use of rhodamine B as a biomarker in a simulated oral vaccine deployment against bovine tuberculosis in white-tailed deer

**DOI:** 10.3389/fvets.2024.1354772

**Published:** 2024-02-13

**Authors:** David Dressel, Kurt C. VerCauteren, Michael J. Lavelle, Nathan P. Snow, Henry Campa

**Affiliations:** ^1^Department of Fisheries and Wildlife, Michigan State University, East Lansing, MI, United States; ^2^USDA APHIS WS National Wildlife Research Center, Fort Collins, CO, United States

**Keywords:** biomarker, bovine tuberculosis, rhodamine B, vaccine delivery, white-tailed deer

## Abstract

**Introduction:**

Free-ranging white-tailed deer (*Odocoileus virginianus*) in northeastern lower Michigan, (United States) are a self-sustaining reservoir for bovine tuberculosis (bTB). Farm mitigation practices, baiting bans, and antlerless deer harvests have been ineffective in eliminating bTB in white-tailed deer and risks to cattle. The apparent prevalence has remained relatively constant in deer, prompting interest among wildlife researchers, managers, and veterinarians for an effective means of vaccinating deer against bTB. The commonly used human vaccine for bTB, Bacillus Calmette Guerin (BCG), is the primary candidate with oral delivery being the logical means for vaccinating deer.

**Materials and methods:**

We developed vaccine delivery units and incorporated the biomarker Rhodamine B before delivering them to deer to assess the level of coverage achievable. Following deployment of Rhodamine B-laden vaccine delivery units on 17 agricultural study sites in Alpena County, MI in Mar/Apr 2016, we sampled deer to detect evidence of Rhodamine B consumption.

**Results and discussion:**

We collected a total of 116 deer and sampled them for vibrissae/rumen marking and found 66.3% (*n* = 77) of the deer collected exhibited evidence of vaccine delivery unit consumption. Understanding the level of coverage we achieved with oral delivery of a biomarker in vaccine delivery units to deer enables natural resource professionals to forecast expectations of a next step toward further minimizing bTB in deer.

## 1 Introduction

Bovine tuberculosis (bTB) is an infectious disease caused by *Mycobacterium bovis* (1) and is maintained in several wildlife reservoirs including European badgers (*Meles meles*) in the United Kingdom (2), France (3), and in the Republic of Ireland (4); brushtail possums (*Trichosurus vulpecula*) in New Zealand (5), cape buffalo (*Syncerus caffer*) in Africa (6); and wild boar (*Sus scrofa*), red deer (*Cervus elaphus*), and fallow deer (*Dama dama*) in Spain (7, 8). In the United States, the wildlife reservoir of bTB is free-ranging white-tailed deer (*Odocoileus virginianus*) (hereafter referred to as ‘deer’) of northeastern lower Michigan (NELM), United States (9). Transmission of bTB between deer and cattle in NELM is a primary concern for wildlife managers, the livestock industry, and the public. Transmission can occur through direct cattle-to-deer contact and indirect contact through shared feed and water (10, 11).

Wildlife managers implemented several methods in attempts to decrease the incidence of bTB in deer and decrease potential risks to cattle. Mitigation methods directed at wildlife have included actions such as exclusionary fences (12), increased antlerless harvest, restrictions on baiting (13), and issuing disease control permits to landowners and United States Department of Agriculture-Wildlife Services (USDA-WS) by Michigan Department of Natural Resources (MDNR) to decrease the incidence of bTB and potential for transmission (14, 15). These strategies have had limited success in reducing the apparent prevalence of bTB thus far. The MDNR established Deer Management Unit 452 (DMU 452) and more recently the expanded DMU 487 to encompass the core area of bTB infection in deer and focus disease management activity. Over the past 15 years the apparent prevalence of bTB in DMU 452 has stalled with minimal fluctuation between 1 and 2% (16, 17). The continued transmission of bTB from deer to cattle and the stalled apparent prevalence has given precedent for seeking novel management strategies to combat bTB.

Oral vaccination of wildlife may be a viable strategy for disease management and is becoming more common for protecting wildlife, livestock, and people against disease transmission. For example, the Oral Rabies Vaccination program targeting raccoons (*Procyon lotor*) distributes nearly 10 million vaccine-laden baits across 18 primarily eastern states of United States annually and has been successful at preventing the spread of rabies (18, 19). Ongoing oral vaccination programs for reservoir hosts of bTB are demonstrating success in reducing incidence of bTB or severity of infection in the European badger in Ireland (20, 21) and the Eurasian wild boar in Spain (22). By combining depopulation efforts with oral vaccination, bTB incidence was significantly reduced in Brushtail possums in New Zealand (23). Experimental oral vaccination of red deer is proving effective and vaccine deployment strategies are being refined in Spain (24, 25).

Researchers have shown the bacillus Calmette-Guerin (BCG) vaccine reduces bTB disease severity in penned white-tailed deer which likely equates to decreased potential to transmit disease (26, 27). Deer that were orally vaccinated with BCG then intratonsilarly challenged with virulent *M. bovis* had reduced gross lesions and a BCG persistence of up to 12 months in lymphoid tissues (26, 27). Additionally, there is some evidence that deer can transmit BCG to unvaccinated deer (28, 29). The efficacy of BCG to be administered orally at scale to deer in NELM provides the capacity to make vaccination against bTB a reality. Although capture and vaccination of deer via injection has been deemed an option, it is labor intensive and costly (30).

Given the availability of a vaccine to inoculate deer against bTB, one primary obstacle for successful oral vaccination was the formulation and field delivery method of a species-specific vaccine delivery unit (VDU) that could be distributed and readily consumed by deer. Oral delivery may be the most cost-effective and feasible method to maximize delivery of a vaccine to a deer population (26). Before a bTB vaccination system can be initiated in free-ranging deer in NELM, understanding the potential coverage of delivery to deer must be investigated.

Rhodamine B (RB) has been used as an effective biomarker for several oral vaccination studies due to (1): the utility of RB as a systemic marker in whiskers and claws (2), the rapid absorption of RB into keratinous tissues (3), the ease of detection of fluorescent bands on whiskers using a fluorescence microscope, and (4) it is commercially available and relatively inexpensive (31). Rhodamine B has proven effective in bait uptake studies of European badgers (32), black-tailed prairie dogs (*Cynomys ludovicianus*) (33), raccoons (34), mountain beavers (*Aplodontia rufa*) (35), stoats (*Mustela ermine*) (36) and wild pigs (*Sus scrofa*) (37).

With current deer harvest rates and the baiting ban in NELM, eradication of bTB is predicted unlikely in the next 30 years. Even with a 100% compliance rate of the baiting ban there is only an 8% chance of reducing the incidence of bTB without implementation of additional strategies (14). However, models have demonstrated a vaccine coverage of 50% in the deer of DMU 452 could achieve an 86% probability of bTB eradication within 30 years (14). Thus, if further reduction or eradication of bTB in NELM is truly desired, additional management strategies must be explored and implemented. By distributing biomarker-laden VDUs to free-ranging deer in NELM it was possible to investigate the potential coverage of vaccination to combat bTB. The primary objective of our evaluation was to quantify the potential coverage of delivering pharmaceuticals orally to free-ranging deer by quantifying uptake of RB in customized VDUs.

## 2 Materials and methods

### 2.1 Study location

We implemented our RB-VDU trial from 7 February 2016 to 26 May 2016 in Alpena County of northeastern lower Michigan, United States Alpena County (439,000 ha) is the northeast county of DMU 452 (147,629 ha), the endemic bTB area with the highest apparent prevalence of bTB in Michigan deer (38). To date, bTB has been identified in 82 cattle herds in the area (39). At the time of the study, there were 189 cattle farms in Alpena County with a total of 8,838 head of cattle (40). One-hundred and eleven (58.7%) of these farms were primarily beef cattle operations and another 37 farms (19.6%) contained mostly dairy cows. Average farm size in Alpena County, United States was 61.1 ha with a total of 458 farms (40). Primary crops produced in Alpena County were hay and grass silage (8,030 ha), soybeans (2,258 ha), corn (2,146 ha) and wheat (1,152 ha) (40). We distributed VDUs on 25 agriculture fields consisting of crops including corn, wheat, alfalfa, or soybeans.

Alpena County consisted of forested land and agriculture lands with deer densities ranging from 10–14 deer/km^2^ (41). Historically, deer density in this area has been as high as 18 deer/km^2^ (42). Average annual temperature in the area was 6.6° C with annual rain and snowfall of 72.5 cm and 175.0 cm, respectively Huey. Elevation ranged from 150-390–m above sea level (43). Well-drained, sandy loam soils comprised much of the landscape and supported a variety of deciduous trees such as aspen (*Populus* spp.) and maple (*Acer* spp.) (44). Lowland conifer stands comprised of conifers such as northern white cedar (*Thuja occidentalis*) and balsam fir (*Abies balsamea*) were an important resource providing deer with thermal cover during winter (44).

Approximately 58% of the deer in this region of Michigan are migratory; most migratory deer (>80%) typically leave winter ranges by 1 May (45). During spring migration, migratory deer typically move to heavily forested areas and away from open-agriculture lands; however, as many as 45% of deer may establish summer ranges near agriculture areas (45). Non-migratory deer in this area tend to establish home ranges in agriculture areas of NELM. Alfalfa fields are an important food resource for deer during the spring, contributing to significant crop loss within 90 m of field edges (46).

### 2.2 Vaccine delivery unit development

Based on previous work in developing VDUs for deer, we determined that an alfalfa and molasses-based matrix would maximize our potential for targeted delivery to deer, while minimizing consumption by non-target species (47). We combined the alfalfa and molasses-based livestock feed (Chaffhaye® Dell City, TX, United States) with Xanthan gum and water in a ribbon mixer to produce a coarse material that could be easily molded. We hand molded each VDU into 17–20-g “bite size” portions to adequately encase the RB-containing capsule while minimizing the potential for spillage which could encourage visitation by nontargets. Using a manual capsule filling machine (CN-100CL, CapsulCN International CO. LTD, Ruian, Zhejiang, China), we inserted 475 mg of RB (7 mg/kg dose for 67.8 kg deer) into 00 size gel capsules (1.17 cm x 2.02 cm), kept in sealed bags at room temperature until needed. This quantity of RB would provide sufficient marking in white-tailed deer, minimize any taste aversion, and was the highest quantity of RB that would physically fit into 00 size capsules. Once in the field, we inserted a single RB capsule into each VDU as we deployed them on agriculture fields. Ingestion of RB-laden VDU by deer causes two staining events (1); the oral (mouth, tongue) and internal cavity (rumen, intestine, and digestive tract) of deer are stained fluorescent pink for 24–36 h after consumption and (2) a fluorescent band appears on deer vibrissae and remains for at least 5 weeks post-consumption (48). The presence of oral, internal, or vibrissae staining allowed us to calculate the percentage of deer that consumed at least one VDU. We recorded total time (min) and cost ($ United States; adjusted to 2023 $) to construct VDUs for the entire process.

### 2.3 Vaccine delivery unit distribution and consumption

We distributed VDUs on 30 agriculture fields on 17 privately owned properties from 6 March 2016 to 26 May 2016. Specific VDU sites were selected using data from road surveys conducted in 2014 by USDA-Wildlife Services during which concentrations of deer were recorded (P. Ryan, Wildlife Biologist, USDA APHIS WS, personal communication). Specific agriculture fields were chosen based on (1), the type of crop grown during the previous year, and (2) anticipated deer activity from conversations with landowners and proximity to vegetation types that would provide deer habitat components. Before VDUs were distributed, all fields considered were monitored with trail cameras (Reconyx, RC60, Holmen, WI, United States) for thawing of snow cover and deer use and abundance from 7 February 2016–6 March 2016. A thawing event was defined as patches of soil and residual crops being exposed in otherwise snow-covered fields resulting from an increase in temperature and exposure to sun. We deployed VDUs when the first thawing event was observed on our VDU grids which coincided with increased deer use.

**Figure 1:**
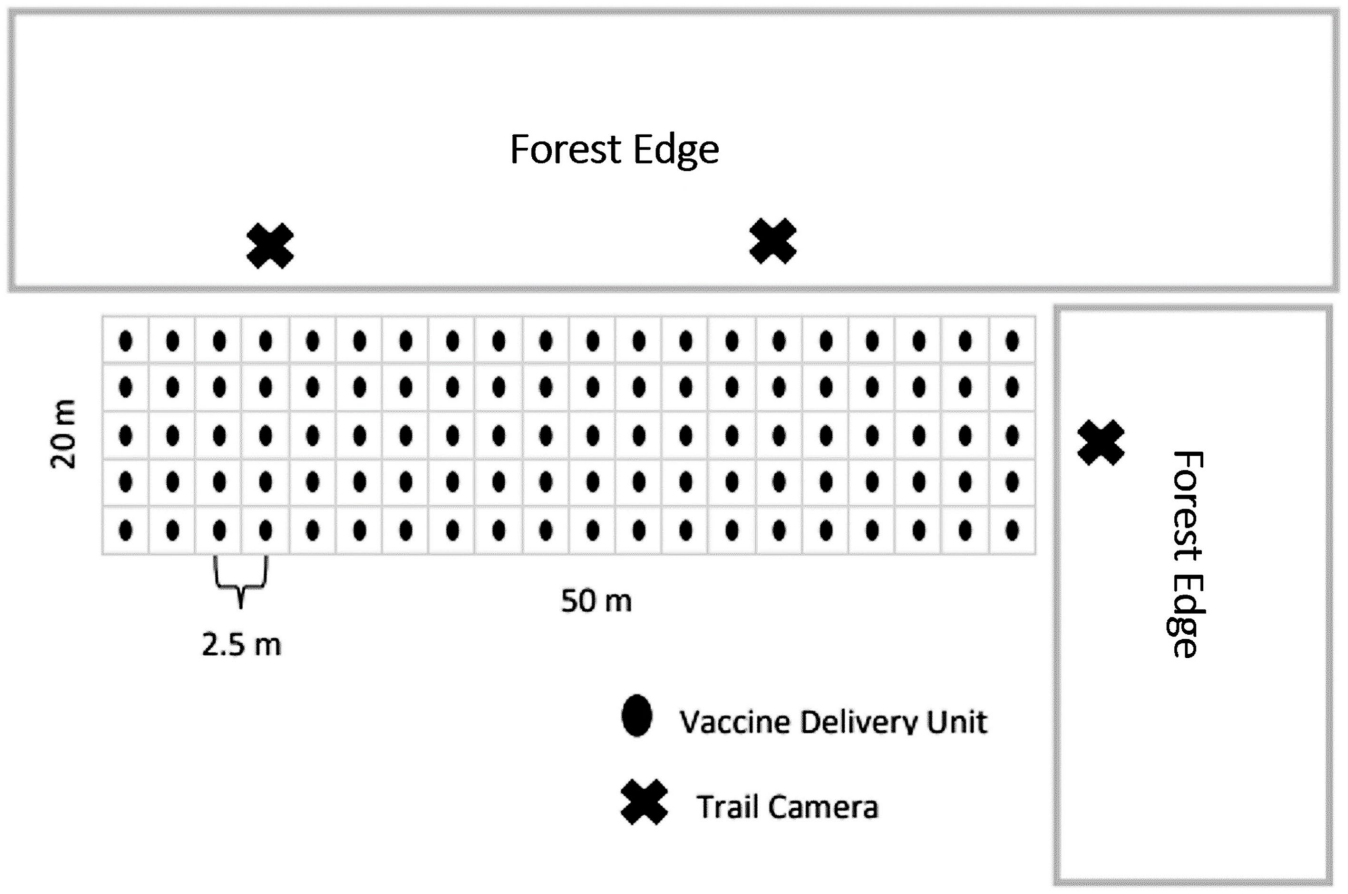
Layout of the 50-m by 20-m, 100-vaccine delivery unit grids placed on agriculture fields next to forest edges in 2016 simulated vaccine deployment against bovine tuberculosis in white-tailed deer (*Odocoileus virginianus*) in northeastern lower Michigan, United States.

We established VDU grids on agricultural fields previously planted to wheat, soybean, alfalfa, or corn, which retained residual crop left after harvest. We determined previously that selecting lowland conifer stands maximized potential for visitation by multiple deer at this time of year (47), thus situated grids adjacent to lowland conifer stands when possible. Each VDU grid consisted of 52.5-m x 12.5-m plots with 100 VDUs spaced 2.5-m apart in grid format (Figure 1). We deployed VDUs for 4–9 consecutive nights with each night that VDUs were distributed being considered a VDU night and used for comparisons of visitation. During the first three VDU nights, we distributed VDUs that did not contain RB to accustom deer to visit grids and consume our VDUs. We checked grids once every 24 h and recorded all VDUs that were missing, assumed eaten and replaced. We recorded the number of VDUs deployed and consumed, paying close attention to whether RB capsules were consumed or left in the field (assumed detected and spit out).

### 2.4 White-tailed deer and non-target visitation

**Figure 2:**
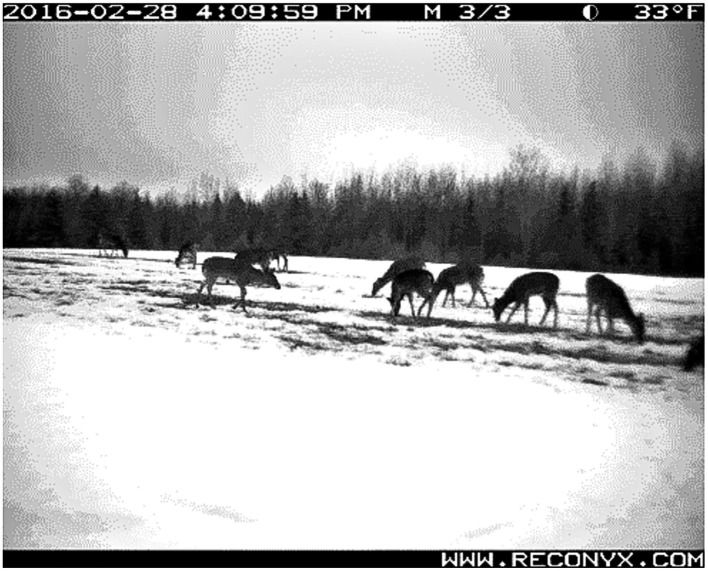
Natural congregation of white-tailed deer (*Odocoileus virginianus*) on a thawed patch of an agricultural field during 2016 simulated vaccine deployment against bovine tuberculosis in white-tailed deer in northeastern lower Michigan, United States.

We installed three trail cameras focused on VDU grids from adjacent field edges and captured motion-activated and time-lapse imagery (1 image every 15 min). Images with the highest number of deer and non-target species in a single frame during a 24-h period were used to determine minimum number of individuals visiting VDU grids (Figure 2). Grid visits were recorded for deer and all non-target species raccoons, skunks (*Mephitis mephitis*), squirrels (*Sciurus* spp.), turkeys (*Meleagris gallopavo*), and eastern cottontail rabbits (*Sylvilagus floridanus*). We compared visitation using trail camera data and the percent of VDU grid nights visited by deer and non-target species.

### 2.5 Biomarker analysis

As early as the last night of VDU distribution, USDA-Wildlife Services began lethally sampling deer on each Rb VDU grid under the direction of MDNR Disease Control Permits. We targeted selection of 10 individual deer per site, but the final number of deer collected was dependent on landowner discretion and success rate. Deer collections were continued each night until our target number of deer was met, or opportunities no longer existed. All deer sampled were first necropsied and visually examined for internal staining of their digestive tract (primarily oral cavity and rumen), confirming RB uptake. Additionally, we collected six maxillary vibrissae (three tactile hairs or “whiskers” from each side of the mouth) from each deer using tweezers and immediately placed into a #7-coin envelope to be evaluated later for detection of fluorescent markings under ultraviolet light (49, 50).

**Figure 3:**
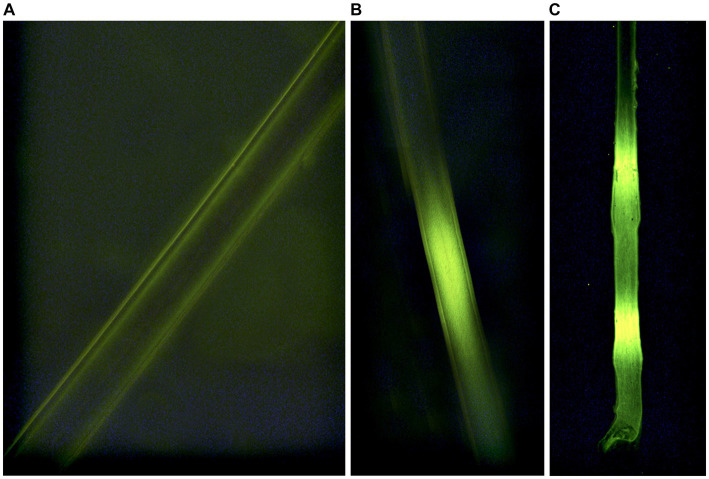
Indications of consumption (**A**-negative; **B,C**-positive) of Rhodamine b in whiskers sampled from white-tailed deer (*Odocoileus virginianus*) during 2016 simulated vaccine deployment against bovine tuberculosis in white-tailed deer in northeastern lower Michigan, United States.

We conducted vibrissae analyses at the USDA/APHIS/Wildlife Services – National Wildlife Research Center (NWRC, Fort Collins, CO, U.S.A.). Vibrissae were mounted on a 75 mm x 25 mm microscope slide (three vibrissae on each slide) using a fluoromount™ aqueous mounting medium. We used a fluorescent microscope (TRITC, Leica, Germany) with a 100 W mercury bulb and RB filter block to identify fluorescent bands on each vibrissae indicating consumption of an RB-laden VDU (Figure 3). All VDU development, deployment, and data collection were reviewed and approved by the Michigan State University Animal Care and Use Committee (AUF # 05/15–084-00; 29 April 2015; Amended 4 January 2016).

### 2.6 Statistical analysis

We examined whether the probability of being marked with RB was influenced by sex of deer using a binomial generalized linear model with the lme4 package (51) in Program R (v 4.2.0, The R Foundation for Statistical Computing). We considered site ID as a random effect to account for site-site variation. We evaluated the parameter estimates and 95% confidence intervals (CIs) of those estimates for non-overlap of zero to indicate statistical and biological differences. We also calculated the model predicted values for the response variables and their 95% CIs for making inferences. We presented the average number of each wildlife species visiting VDU grids and examined for non-overlap of standard errors, suggesting statistical difference in overall visitation.

## 3 Results

### 3.1 Vaccine delivery unit distribution and consumption

We distributed a total of 7,080 VDUs to free-ranging deer in NELM across 30 VDU grids on 17 sites during the 2016 field season. Overall, 3,279 non-RB VDUs were distributed and 1,878 (57.2%) were consumed. A total of 3,801 VDUs containing RB were distributed of which 2,101 (55.3%) were consumed. However, deer rejected 34.64% of the RB capsules; evidenced by the consumption of the VDU and not the RB capsule.

### 3.2 White-tailed deer and non-target visitation

**Figure 4:**
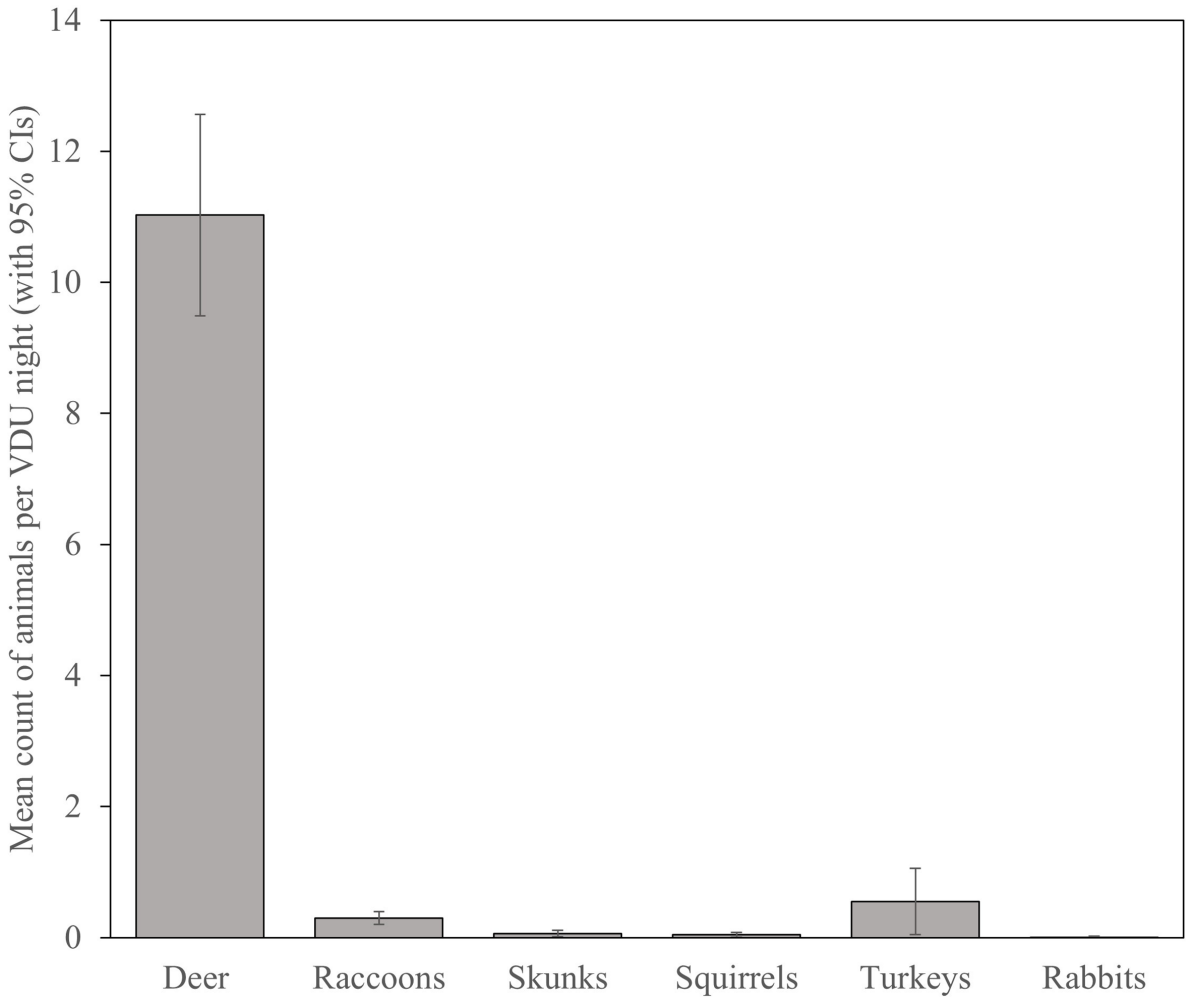
Mean count (with 95% CIs) of white-tailed deer (*Odocoileus virginianus*) and non-target species visiting simulated vaccination sites and potentially consuming vaccination delivery units during 2016 evaluation of oral vaccine deployment against bovine tuberculosis in white-tailed deer in northeastern lower Michigan, United States.

With 113 VDU nights recorded from 6 March to 28 April, we calculated a minimum average of 11.03 (SE = 0.78) deer visiting sites per 24-h (Figure 4), though the highest number of deer per 24-h photographed on a single site was 45 deer on 5 April 2016. Turkeys and raccoons were the second and third most prevalent species visiting sites though averaged only 0.55 (SE = 0.26) and 0.30 (SE = 0.05) per night, respectively. Documented visitation by turkeys was limited to 30% (5 of 17) of sites with 87% (54 of 62) observed on one site with a flock of as many as 22 birds. Visitation by raccoons was more widespread across 76% (13 of 17) of sites, though were lower in number with a maximum of 5 observations on three sites. Skunks, squirrels, and rabbits were observed, though very rarely, on VDU grids.

### 3.3 Biomarker analysis

Overall, we sampled 116 deer from 17 sites. The number of deer sampled per site ranged from 1 to 13. We observed that 77 (66.3%) of the deer sampled were marked with RB (range = 0–100%). Of the 77 deer marked, 6 were identified RB positive by internal staining and 71 were identified RB positive by vibrissae marking. Although when excluding sites with ≤3 deer sampled, the percent marked ranged from 20–100%. We found there was no difference in the probability of being marked between males and females (*β* = 0.02, 95% CI = −0.80–0.87). Model predictions indicated that males had a 0.66 probability (95% CI = 0.50–0.80), and females had a 0.66 (95% CI = 0.55–0.76) probability of being marked.

### 3.4 Vaccine delivery unit development and cost

The total time to produce 800 VDUs (average VDUs produced from a single 22.68 kg bag of dry product) with RB was 200 min. Time estimates included encapsulating RB, mixing ingredients, and forming VDUs by hand. Based on average consumption across sites, the overall cost of producing and deploying VDUs was $654/site.

## 4 Discussion

We demonstrated that it was possible to deliver pharmaceuticals to the majority of free-ranging white-tailed deer visiting our selected agricultural fields in late winter/early spring in NELM. Specifically, we found the alfalfa/molasses VDUs we developed were sufficiently palatable to be sought out and readily consumed by deer. Our distribution strategy utilizing single VDUs dispersed across an elongated rectangular grid design facilitated delivery to individuals within groups of deer while minimizing nose-to-nose contact and associated potential for disease transmission. By locating our grids in agricultural fields and adjacent to lowland conifer stands, deer appeared to encounter them during daily movements typical of late-winter and early spring. Using RB, we successfully confirmed consumption of ≥1 RB-laden VDUs in 66.3% of the 116 deer sampled. This is 16.3% above the 50% vaccination rate in simulation models needed to achieve an 86% probability of eradication of bTB in 30 years if used in conjunction with other ongoing management strategies (14).

By timing the initiation of our VDU deployment during initial thawing events and winter break-up (6 March 2016), we benefitted from seasonal concentrations of deer. Deer in Michigan demonstrate high site fidelity to yarding areas associated with lowland conifer stands (52, 53) and as environmental conditions permit, (i.e., decrease in snow cover and depth) deer leave their associated yarding areas to search for spring foods (53). As such, an increase in deer abundance on agriculture fields occurs during this time in NELM and may be a condition of the proximity of agriculture lands to lowland conifer stands (45). Deer metabolism also begins to increase with the initiation of spring (March and April) (54), resulting in dispersal to feed on agriculture waste grain and alternative agricultural foods that provide needed nutritional components. By deploying VDUs early in the winter break-up period (March and April), as opposed to May and June, we observed relatively more deer on our VDU grids compared to a 2015 trial (47). Later, deer disperse, targeting newly sprouting vegetation, especially in aspen/birch stands and upland mixed forest stands to meet their spring and summer life requisites (44, 55). These seasonal dispersals may suggest the appropriate time to cease targeted oral vaccinations, as fewer deer will encounter VDU grids, and food preferences and demands will likely have changed.

The timing of our simulated vaccination also benefitted from seasonally reduced activity and visitation by most non-target species except for occasional turkeys (47). Consumption of VDUs intended for deer has the potential to hinder the delivery of VDUs to all visiting deer, though complete consumption in a single night was never an issue. Ongoing monitoring with cameras throughout the deployment process could inform the number of VDUs needed to maximize coverage of deer visiting. Additionally, delivery of vaccine-laden VDUs over multiple nights, with monitoring between nights would alert VDU deployment crews to situations in which all VDUs were consumed, suggesting an insufficient number of VDUs and need to increase numbers being delivered.

Wildlife managers must take into consideration the efficacy of the methods and the cost associated with an oral vaccination of deer in NELM. The alfalfa/molasses VDU we developed and used was relatively inexpensive to produce. With an average cost to produce and deploy alfalfa/molasses VDUs (without vaccine) of $654 per site (for 6 days), the use of this oral vaccination strategy on the entirety of DMU 452 is a real possibility. The cost of expanding this oral vaccination across DMU 452 (1,476 km^2^) would need to take into consideration the number of VDUs to distribute, the cost of the BCG vaccine, the spatial scale at which distribution would occur and the cost of specialized training needed to handle the BCG vaccine. The cost of an oral vaccination across DMU 452 would likely be substantially lower than the estimated cost for other proposed management strategies of vaccine delivery (i.e., trap/vaccinate methods, $1.5 million annually) (30). We are aware that the cost estimate may increase when BCG is added to the VDUs but may still be cost effective at 0.36 to 0.67 cents/dose (42) (M. Palmer, Veterinary Medical Officer, USDA ARS National Animal Disease Center).

The relatively low cost of production, relatively high consumption rates by deer, and minimal non-target visitation makes the alfalfa/molasses VDU a suitable candidate to deliver the BCG vaccine to free-ranging deer adjacent to lowland conifer, then shifting to aspen/birch stands during winter break-up in NELM. With the use of a biomarker (RB) we demonstrated that by targeting deer on agriculture fields during winter break-up, it may be possible to vaccinate the targeted ≥50% of deer on the landscape. It is also possible for wildlife managers and others to expedite the development of VDUs and the deployment strategy. By mixing larger quantities of ingredients and with aid of off-road vehicles and mechanical feeders, managers may be able to decrease the time needed to distribute VDUs. Further research should evaluate the efficacy of BCG vaccine insertion into these VDUs and the viability of distributing BCG to deer of NELM. Developing this vaccination strategy has shown it may be a cost-effective strategy to vaccinate when compared to other labor-intensive strategies i.e., trap and vaccinate (30); and could be a significant contribution to ongoing wildlife disease mitigation strategies implemented in the area.

## 5 Conclusion

The development of our alfalfa/molasses VDU and associated delivery strategy may be the most scalable and effective method for vaccinating deer in NELM against bTB. Initiating an oral vaccination program during winter break-up would help maximize the number of deer that encounter and consume VDUs. Further, initial vaccination efforts should target using agriculture fields adjacent to lowland conifer stands at the end of winter-early spring (March). If efforts extend into late spring (May), a shift toward agriculture fields near aspen/birch stands would follow shifts in habitat use by deer. This continuous and adaptive strategy would allow the vaccination effort to target those deer with high site fidelity to lowland conifer stands and migratory deer moving to spring food resources in late spring. Bovine tuberculosis is a pervasive issue in NELM and the continued spillover into cattle poses great economic and social consequences for many stakeholders. The 66.3% coverage of free-ranging deer that we achieved exceeds the previously stated vaccination rate of 50% needed to maximize the probability of eradication of bTB in 30 years. Our proposed vaccination strategy could be an additional management tool to combat bTB in NELM and further progress toward eradicating the disease.

## Data availability statement

The raw data supporting the conclusions of this article will be made available by the authors, without undue reservation.

## Ethics statement

The animal study was approved by Michigan State University Animal Care and Use Committee. The study was conducted in accordance with the local legislation and institutional requirements.

## Author contributions

DD: Conceptualization, Data curation, Formal analysis, Investigation, Methodology, Writing – original draft, Writing – review & editing. KV: Conceptualization, Funding acquisition, Methodology, Project administration, Resources, Supervision, Writing – review & editing. ML: Conceptualization, Investigation, Methodology, Writing – review & editing. NS: Formal analysis, Writing – review & editing. HC: Conceptualization, Funding acquisition, Methodology, Project administration, Resources, Supervision, Writing – review & editing.
